# Instituting CDC Draft Guidelines for Prophylaxis of Neonatal Group B Streptococcal Invasive Disease

**DOI:** 10.1155/S1064744995000664

**Published:** 1995

**Authors:** Marvin S. Amstey

**Affiliations:** Department of Obstetrics and Gynecology University of Rochester School of Medicine Genesee Hospital Rochester Near York USA


Infectious Diseases in Obstetrics and Gynecology 3:221 (1995)
© 1996 Wiley-Liss, Inc.

### Instituting CDC Draft Guidelines for Prophylaxis of Neonatal Group B Streptococcal Invasive Disease

Prior to the circulation of the CDC draft guidelines and the discussion of these at the Infectious Disease Society for Obstetrics and Gynecology (IDSOG) in 1995, the attending staff at Genesee Hospital followed 5 different protocols for using prophylactic antibiotics to prevent group B streptococcal (GBS) disease in the newborn. With a planned education approach for the attending and hospital staff, we were able to accomplish virtually 100% acceptance and compliance with the CDC draft guidelines.

The first step in this process was to present the history of the problem, the computer model from the Rouse paper, and the proposed CDC guidelines. Next, we had one-on-one discussions with the microbiology staff at our hospital regarding the benefits of broth incubation of the culture specimen prior to plating on a selective medium. The third step was to meet with the personnel on the labor floor and the nurses in the nursery and provide them with the same education, stressing the importance of follow-up of all cultures at 35–37 weeks gestation.

A system was set up whereby the clerk on the labor floor would call the microbiology laboratory whenever a patient was admitted to the labor floor. In addition, the microbiology laboratory faxed copies of all GBS-only genital culture reports each day to the labor floor where they were alphabetically filed, making the reports available to the house staff physicians when they admitted their patients.

Inherent in this system was the need for every attending physician to follow the guidelines. In our case, the guidelines were adhered to by 23 of 24 attending physicians. Also inherent in the system was the requirement that all attending physicians use the same laboratory, which was adhered to by all 24 attending physicians. Lastly, the microbiology laboratory kept records of direct plating vs. broth enhancement, with a 15% increase in sensitivity from the broth enhancement in the first 4 months of this activity.

In summary, a 3-month education system including an introduction to the new CDC draft guidelines for preventing neonatal GBS sepsis resulted in a uniform system of activity by the attending staff at our hospital. This system should enable us, over a short time, to perform an outcome analysis of the protocol's effectiveness in reducing GBS disease in the newborn. Our experience is a good example of how the education and involvement of all vested interests can make the introduction of a potentially useful and cost-effective protocol possible.

*Marvin S. Amstey*
*Department of Obstetrics and Gynecology*
*University of Rochester School of Medicine*
*Genesee Hospital*
*Rochester, New York*

Editorial

Received April 1, 1996

Accepted April 3, 1996

